# Significant Fibrosis Is Not Rare in Chinese Chronic Hepatitis B Patients with Persistent Normal ALT

**DOI:** 10.1371/journal.pone.0078672

**Published:** 2013-10-25

**Authors:** Baolin Liao, Zhanhui Wang, Siwei Lin, Ying Xu, Junqing Yi, Min Xu, Zuxiong Huang, Ying Zhou, Fuchun Zhang, Jinlin Hou

**Affiliations:** 1 Hepatology Unit and Key Lab for Organ Failure Research, Nanfang Hospital, Southern Medical University, Guangzhou, China; 2 Department of Hepatology, Guangzhou No.8 People’s Hospital, Guangzhou Medical University, Guangzhou, China; 3 Department of Third Internal Medicine, Yuexiu District Traditional Chinese Medicine Hospital, Guangzhou, China; Yonsei University College of Medicine, Korea, Republic Of

## Abstract

**Background:**

Limited studies have been done on chronic hepatitis B (CHB) patients defined according to the latest Asian-Pacific Association for the Study of the Liver guideline with liver histology by a large sample size.

**Methods:**

We retrospectively evaluated liver histological characteristics on a cohort of consecutive treatment-naive CHB patients with persistent normal alanine aminotransferase (PNALT) or elevated ALT from May 2005 to October 2011. Histological assessment was based on the Metavir scoring system, significant abnormality was defined as necroinflammation grade ≥A2 and/or fibrosis stage ≥F2.

**Results:**

A total of 675 CHB patients were recruited, including 516 HBeAg-positive and 159 HBeAg-negative patients. In HBeAg-positive patients, significant fibrosis was found 49.4% (42/85) in PNALT, 69.8% (88/126) in ALT 1-2×upper limit normal (ULN) and 81.6% (249/305) in ALT>2×ULN group, respectively. In HBeAg-negative patients, significant fibrosis was found 30.9% (17/55) in PNALT, 73.3% (33/45) in ALT 1-2×ULN and 94.9% (56/59) in ALT>2×ULN group, respectively. HBeAg-positive patients with PNALT over 30 years old had a higher frequency of significant fibrosis than those under 30 years old (87.5% vs. 45.5%, P = 0.058). Multivariate logistic regression analysis indicated increasing age (P = 0.012), higher aspartate aminotransferase (AST) (P < 0.001) and lower HBV DNA (P < 0.001) were associated with significant necroinflammation, while higher AST (P < 0.001), lower albumin (P = 0.027) and HBV DNA (P = 0.004) were associated with significant fibrosis in HBeAg-positive patients with elevated ALT. Higher AST was associated with significant necroinflammation in HBeAg-negative patients with elevated ALT (P = 0.009).

**Conclusions:**

Significant fibrosis is not rare in Chinese CHB patients with PNALT, especially HBeAg-positive patients over 30 years old.

## Introduction

Hepatitis B virus (HBV) infects 400 million people worldwide, and more than 75% of them reside in the Asian-Pacific area [1]. Chronic hepatitis B (CHB) is the major risk factor for cirrhosis, end-stage liver disease and hepatocellular carcinoma (HCC) [2,3]. Moreover histological abnormalities are correlated with long-term risk [4]. So correctly assessing liver histological abnormalities is very important in evaluating disease severity and management. Although much progress has been made in using transient elastography (TE) to assess liver fibrosis, disadvantages should not be ignored for it could not indicate inflammatory activity grade，lower fibrosis stages (F0 - F2) precisely and is influenced by elevated alanine aminotransferase (ALT) [5]. Additionally, TE has not been installed in most hospitals in China yet. Therefore liver biopsy remains the gold standard in assessing liver histological abnormalities though its limitations do exist [6,7], while TE could be used as a complementary tool [8].

The 2012 Asian-Pacific Association for the Study of the Liver (APASL) guideline recommended liver biopsies should be considered in viremic CHB patients over 40 years old, especially those with high normal or minimally raised ALT [8]. The American Association for the Study of Liver Diseases (AASLD) guideline proposed a similar opinion [9]. However, the 2012 European Association for the Study of the Liver (EASL) guideline suggested beside patients with fluctuated ALT, liver biopsies or even therapy should be considered in immune tolerant patients over 30 years old [10]. Opinions on what kinds of patients should be considered for liver biopsy, especially those with normal ALT, among these guidelines are a little different. Previous researches on patients’ liver histological abnormalities were limited by either small sample size or only concentrated on patients with elevated ALT [11–15]. Furthermore, EASL guideline suggested future research should focus on unresolved issues including indications for treatment in immune tolerant patients, and HBeAg-negative patients with level of HBV DNA below 20,000 IU/mL (100,000 copies/mL) [10]. So in this study, we aim at comprehensively evaluating the characteristics of histological abnormalities by a large population of Chinese CHB patients with persistent normal ALT (PNALT) or elevated ALT.

## Methods

### Patients

We reviewed all CHB patients who were hospitalized in Guangzhou No.8 People’s hospital from May 2005 to October 2011. The indications of liver biopsy were as follows：patients with PNALT wish fully assessing the severity of liver fibrosis and inflammation voluntarily and getting further treatment advice afterwards; patients with elevated ALT when there was clinical indication of assessing the severity of liver fibrosis and inflammation prior to antiviral therapy. A total of 3328 consecutive patients who also had a liver biopsy were screened. The inclusion criteria were as follows: (1) HBsAg positive for at least the previous 6 months, (2) HBeAg-positive patients with PNALT and HBV DNA >100,000 copies/mL or HBeAg-negative patients with PNALT and HBV DNA <100,000 copies/mL, or patients with elevated ALT. PNALT was defined by having at least three ALT values equal to or less than 40 U/L every 6-12 months apart with the observation periods from 18-36 months and no elevated ALT at any time points prior to liver biopsy. While the inclusion criteria of HBV DNA level below 100,000 copies/mL in HBeAg-negative patients with PNALT was determined according to the APASL guideline and responding to the unresolved issues in the EASL guideline. The exclusion criteria were as follows: (1) hepatitis C or D or human immunodeficiency virus coinfection, (2) evidence of liver disease because of other etiology, (3) use of hepatotoxic drugs or regular consumption of alcohol, (4) received antiviral (HBV) therapy or any liver functional protection therapy to alleviate the hepatic inflammation before, and (5) patients had less than three normal ALT values prior to the biopsy. 

The study protocol was conducted within the guidelines of the 1975 Declaration of Helsinki, and was approved by the ethics committee of Guangzhou No.8 People’s hospital. Written informed consent was obtained from all subjects. 

### Serological, biochemical and HBV DNA assay

Biochemical tests and complete blood cell counts were performed using routine automated analyzers. HBV and other serological markers were detected by chemiluminescent enzyme immunoassay (Abbott Laboratories, Chicago, IL, USA). Serum ALT, AST, albumin (ALB) and prothrombin activity (PTA) levels were determined by commercial kits. The ULN of ALT and AST were 40 U/L for both male and female. The level of HBV DNA was measured by real-time PCR with a lower detection limit of 1000 copies/mL (DaAn Gene Co, China).

### Liver biopsy and histology assessment

Liver biopsies were obtained using a 16G core aspiration needle, with a biopsy length at least 1.5 cm and contained 6 portal tracts or more. Biopsies were fixed, paraffin-embedded, and stained with hematoxylin and eosin for morphological evaluation and Masson’s trichrome stain for assessment of fibrosis. The pathologist reviewing all the biopsy specimens was blinded to the biochemical and virologic results of the patients. Liver biopsies were scored using the Metavir scoring system for both necroinflammation grade and fibrosis stage [16]. Significant histological abnormality was defined as necroinflammation grade ≥A2 and/or fibrosis stage ≥F2.

### Statistical analysis

All data were analyzed using the statistical package SPSS (version13.0; SPSS, Inc., Chicago, IL). Results were given as mean±SD or no.(%) of patients. Levels of HBV DNA were transferred to log_10_ copies/mL. Chisquare was used for categorical variables. Mann-Whitney or Kruskal-Wallis test was used for similar comparison of nonparametric data. A univariate analysis was firstly performed to determine if any clinical variables were associated with significant abnormalities. Multivariate logistic regression was then used to determine whether the identified clinical variables from above were independent risk factors associated with significant abnormalities. Two tailed P-value of <0.05 was considered statistically significant. 

## Results

**Figure 1:**
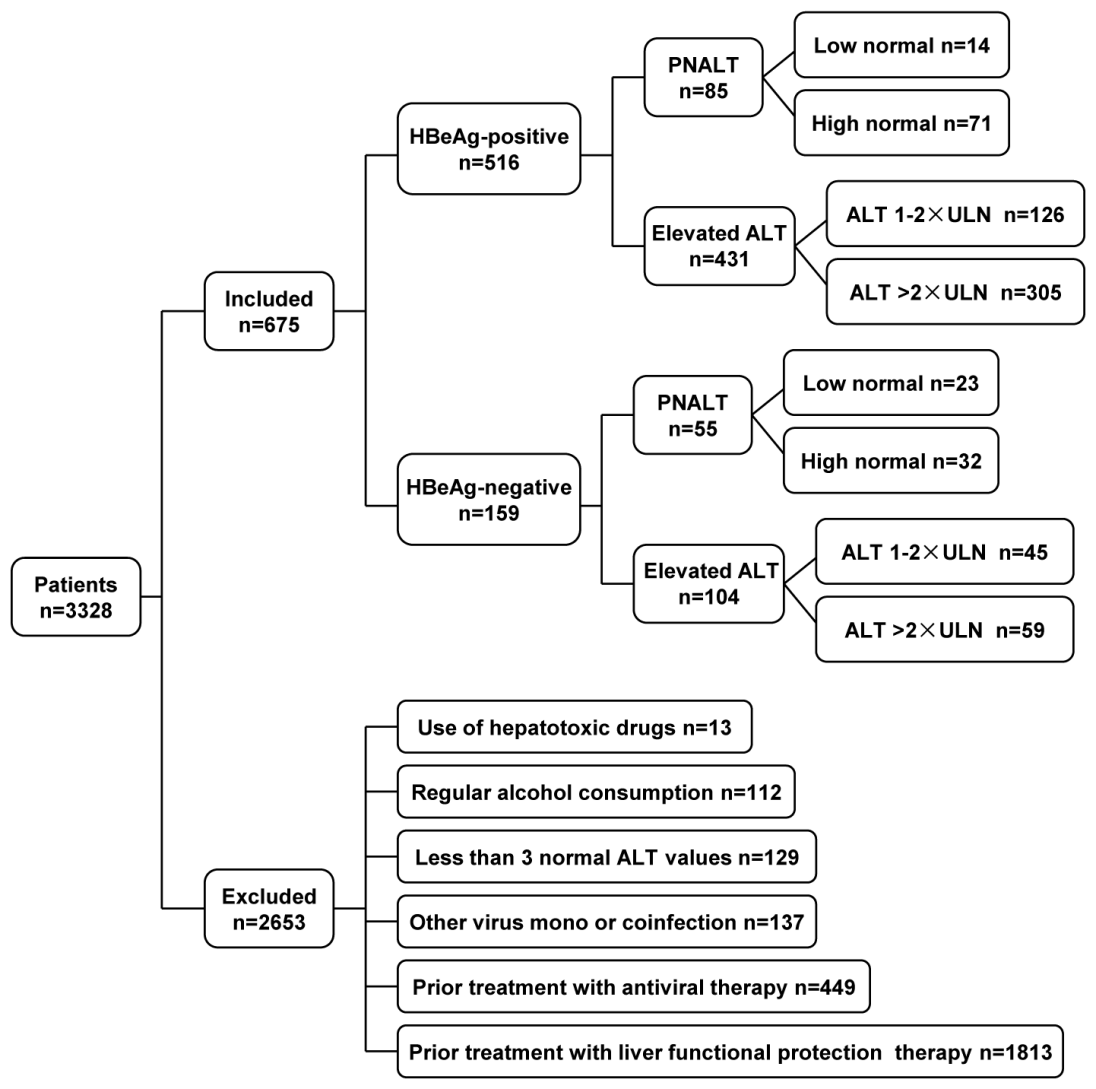
Flow chart of study design. We screened 3328 patients with liver biopsy from May 2005 to October 2011 and 675 met our inclusion criteria, 2653 patients were excluded. Among the included patients, 516 were HBeAg-positive patients (85 patients with PNALT and 431 with elevated ALT) and the remaining 159 were HBeAg-negative patients (55 with PNALT and 104 with elevated ALT).

Among the 3328 patients screened, 675 met the inclusion criteria including 516 HBeAg-positive and 159 HBeAg-negative patients. In HBeAg-positive patients, 85 had PNALT and 431 had elevated ALT. In HBeAg-negative patients, 55 had PNALT and 104 had elevated ALT. The details of included and excluded patients are shown in Figure 1.

### Liver histological characteristics of patients

#### HBeAg-positive patients

**Figure 2:**
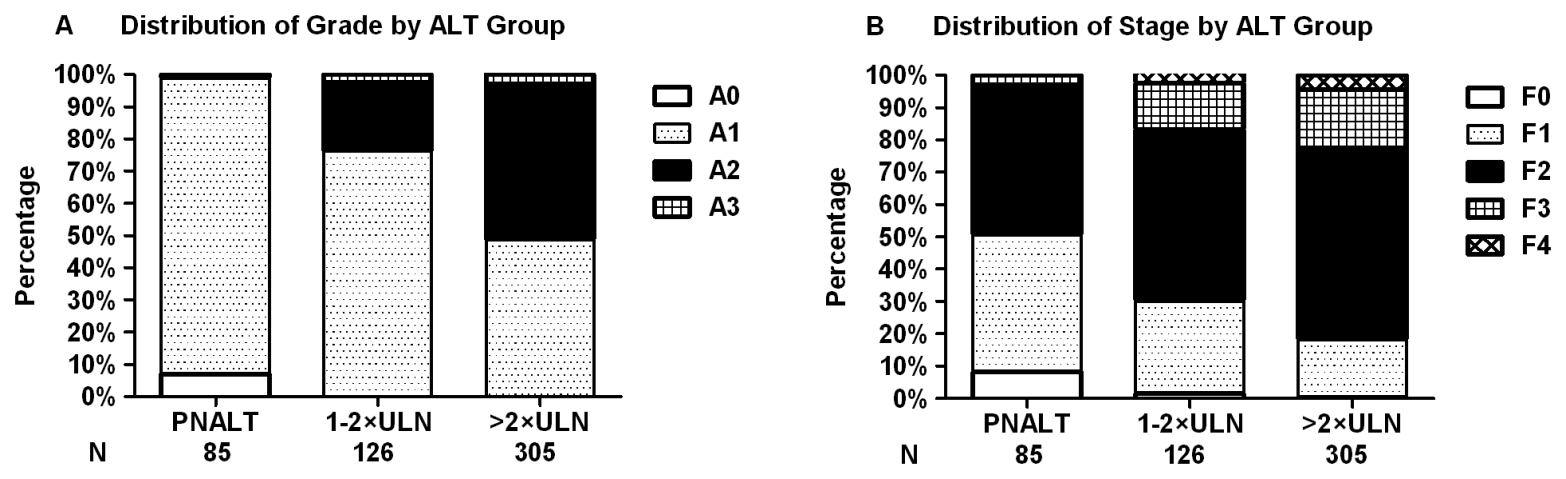
Necroinflammation grade and fibrosis stage in HBeAg-positive patients. (A) Necroinﬂammation grade in HBeAg-positive patients. Significant necroinflammation (≥A2) was found 1.2%, 23.8% and 51.1% in PNALT, ALT 1-2×ULN and >2×ULN group, respectively. (B) Fibrosis stage in HBeAg-positive patients. Significant fibrosis (≥F2) was found 49.4%, 69.8% and 81.6% in PNALT, ALT 1-2×ULN and >2×ULN group, respectively. Significant histological abnormalities in >2×ULN group were much higher than those in PNALT or ALT 1-2×ULN group (both P < 0.001).

The demographic profiles and parameters of HBeAg-positive patients are shown in Table 1. Patients with elevated ALT were mainly older males, and had lower levels of HBV DNA than patients with PNALT. The distribution of significant necroinflammation was 1.2% (1/85) in PNALT, 23.8% (30/126) in ALT 1-2×ULN and 51.1% (156/305) in ALT >2×ULN group, while significant fibrosis was 49.4% (42/85) in PNALT, 69.8% (88/126) in ALT 1-2×ULN and 81.6% (249/305) in ALT >2×ULN group, respectively. Frequencies of histological abnormalities in patients with ALT >2×ULN were much higher than in those of patients with PNALT or ALT 1-2×ULN (both P < 0.001) (Figure 2). According to the latest APASL guideline [8], patients with PNALT were further stratified into low normal (≤0.5×ULN) and high normal (0.5-1×ULN) subgroups based on the pre-biopsy ALT values. No differences were found in liver histological significant necroinflammation (P = 1.000) and significant fibrosis (P = 0.527) between low normal and high normal ALT subgroups. Patients with PNALT were also stratified by age of 30 to verify the EASL’s suggestion. Results indicated frequency of significant necroinflammation were similar between subgroups (P = 1.000), but older patients had a higher frequency of significant fibrosis than younger patients (P = 0.058) with the difference almost reaching statistical significance. (Table 2)

**Table 1:** Demographic and clinical characteristics of HBeAg-positive and HBeAg-negative patients.

	HBeAg-positive			HBeAg-negative		
Patients characteristics	PNALT (n = 85)	Elevated ALT (n = 431)	P Value	PNALT (n = 55)	Elevated ALT (n = 104)	P Value
Age	23.8±6.7	27.8±7.3	<0.001	35.4±7.2	33.5±9.1	NS
Male	45 (52.9%)	283 (65.7%)	0.026	28 (50.9%)	82 (78.8%)	<0.001
PLT (×10^9^/L)	212.3±57.5	194.8±54.4	0.012	211.1±67.8	182.7±51.7	0.005
PTA (%)	103.4±17.5	98.6±21.8	0.017	106.2±18.9	97.9±22.0	0.007
ALB (g/L)	45.3±3.5	44.0±4.2	0.013	44.5±4.2	44.1±4.0	NS
ALT (U/L)	27.0±6.7	143.2±78.3	<0.001	23.8±8.0	119.6±80.2	<0.001
≤0.5× ULN	14 (16.5%)	( - )		23 (41.8%)	( - )	
0.5-1 × ULN	71 (83.5%)	( - )		32 (58.2%)	( - )	
1-2 × ULN	( - )	126 (29.2%)		( - )	45 (43.3%)	
>2 × ULN	( - )	305 (70.8%)		( - )	59 (56.7%)	
AST (U/L)	24.4±4.5	90.4±52.9	<0.001	23.9±5.7	78.9±58.0	<0.001
HBV DNA (log_10_ copies/mL)	7.55±0.70	6.95±1.09	<0.001	3.21±0.47^*^	5.54±1.27	<0.001

Parameters are expressed as mean±SD or number (%)

PNALT, persistent normal ALT; PLT, platelet; PTA, prothrombin activity; ALB, albumin; ALT, alanine aminotransaferase; AST, aspirate aminotransferase; ULN, upper limit of the normal range; NS, not significant

The normal range of ALT and AST are 5-40 U/L, PLT is 100-300 ×10^9^/L, ALB is 35-55 g/L

* 21 subjects with undetectable HBV DNA

**Table 2:** Characteristics of HBeAg-positive and HBeAg-negative patients with PNALT stratified by ALT and age.

	HBeAg-positive						HBeAg-negative					
	ALT≤0.5× ULN	ALT 0.5- 1×ULN	P Value	Age≤30	Age>30	P Value	ALT≤0.5× ULN	ALT 0.5- 1×ULN	P Value	Age≤40	Age>40	P Value
	(n = 14)	(n = 71)		(n = 77)	(n = 8)		(n = 23)	(n = 32)		(n = 46)	(n = 9)	
Age	23.3±3.7	23.6±6.5	NS	( - )	( - )	( - )	36.2±7.7	34.8±6.8	NS	( - )	( - )	( - )
Male	5 (35.7%)	40 (56.3%)	NS	41 (53.2%)	4 (50.0%)	NS	6 (26.1%)	22 (68.8%)	0.002	24 (52.2%)	4 (44.4%)	NS
PLT (×10^9^/L)	211.0±59.0	212.5±57.7	NS	214.3±58.7	192.5±43.3	NS	227.0±83.9	199.8±52.0	NS	208.8±64.1	223.2±88.0	NS
PTA (%)	102.2±12.9	103.6±18.4	NS	103.1±17.2	105.8±22.0	NS	106.5±17.4	106.0±20.2	NS	104.6±18.1	114.2±22.0	NS
ALB (g/L)	43.8±3.2	45.6±3.5	NS	45.4±3.4	43.3±3.8	NS	44.4±3.8	44.7±4.5	NS	44.9±4.0	42.6±4.5	NS
ALT (U/L)	( - )	( - )	( - )	27.0±6.8	27.5±6.3	NS	( - )	( - )	( - )	23.4±7.8	25.8±8.9	NS
AST (U/L)	21.6±2.9	24.9±4.6	0.004	20.7±4.0	22±4.0	NS	20.8±4.0	26.1±5.8	<0.001	23.7±5.4	25.2±7.4	NS
HBV DNA (log_10_ copies/mL)	7.25±0.67	7.61±0.69	NS	7.55±0.71	7.58±0.62	NS	3.17±0.43	3.25±0.50	NS	3.21±0.46	3.20±0.52	NS
Undetectable HBV DNA	( - )	( - )	( - )	( - )	( - )	( - )	9 (39.1%)	12 (37.5%)	NS	18 (39.1%)	3 (33.3%)	NS
Inflammation grade												
<A2	14 (100%)	70 (98.6%)	NS	76 (98.7%)	8 (100%)	NS	23 (100%)	27 (84.4%)	NS	41 (89.1%)	9 (100%)	NS
≥A2	0 (0%)	1 (1.4%)		1 (1.3%)	0 (0%)		0 (0%)	5 (15.6%)		5 (10.9%)	0 (0%)	
Fibrosis stage												
<F2	6 (42.9%)	37 (52.1%)	NS	42 (54.5%)	1 (12.5%)	0.058	19 (82.6%)	19 (59.4%)	NS	32 (69.6%)	6 (66.7%)	NS
≥F2	8 (57.1%)	34 (47.9%)		35 (45.5%)	7 (87.5%)		4 (17.4%)	13 (40.6%)		14 (30.4%)	3 (33.3%)	

Parameters are expressed as mean±SD or number (%)

PNALT, persistent normal ALT; PLT, platelet; PTA, prothrombin activity; ALB, albumin; ALT, alanine aminotransaferase; AST, aspirate aminotransferase; ULN, upper limit of the normal range; NS, not significant

The normal range of ALT and AST are 5-40 U/L, PLT is 100-300 ×10^9^/L, ALB is 35-55 g/L

#### HBeAg-negative patients

**Figure 3:**
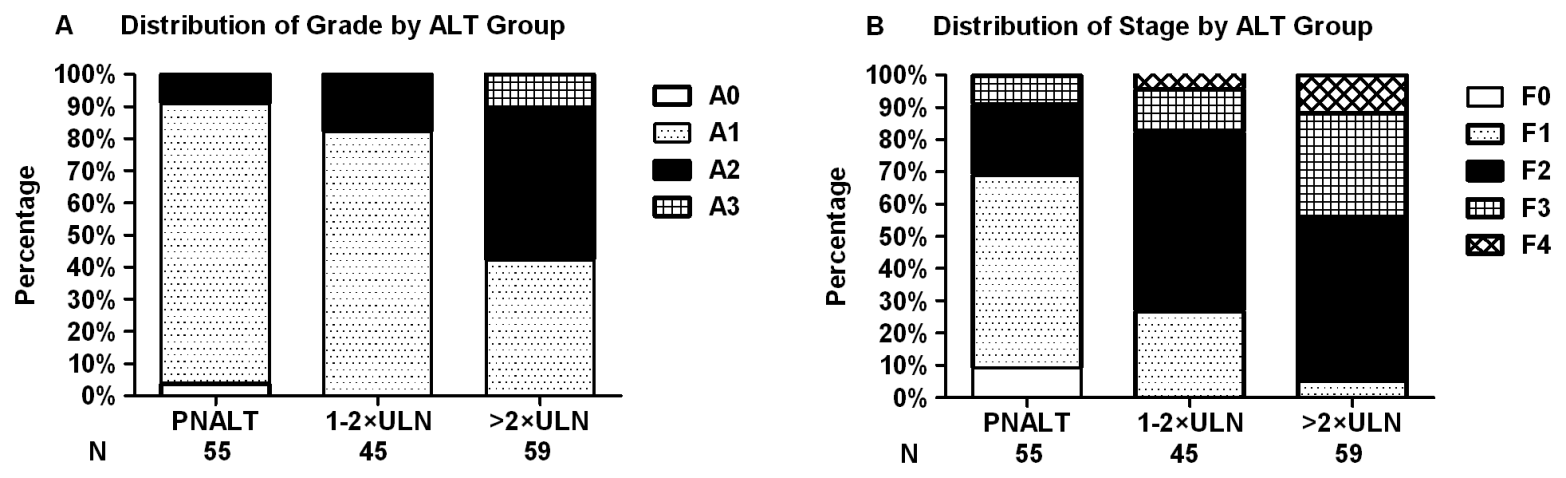
Necroinflammation grade and fibrosis stage in HBeAg-negative patients. (A) Necroinﬂammation grade in HBeAg-negative patients. Significant necroinflammation (≥A2) was found 9.1%, 17.8% and 57.6% in PNALT, ALT 1-2×ULN and >2×ULN group, respectively. (B) Fibrosis stage in HBeAg-positive patients. Significant fibrosis (≥F2) was found 30.9%, 73.3% and 94.9% in PNALT, ALT 1-2×ULN and >2×ULN group, respectively. Significant histological abnormalities in >2×ULN group were much higher than those in PNALT or ALT 1-2×ULN group (both P < 0.001).

The baseline demographic profile and parameters of HBeAg-negative patients are shown in Table 1. The distribution of significant necroinflammation was 9.1% (5/55) in PNALT, 17.8% (8/45) in ALT 1-2×ULN and 57.6% (34/59) in ALT >2×ULN group, while significant fibrosis was 30.9% (17/55) in PNALT, 73.3% (33/45) in ALT 1-2×ULN and 94.9% (56/59) in ALT >2×ULN group, respectively. Histological abnormalities in patients with ALT >2×ULN were much higher than in those of patients with PNALT or ALT 1-2×ULN (both P < 0.001) (Figure 3). When HBeAg-negative patients with PNALT were stratified by ALT, no differences were found in liver histological significant necroinflammation (P = 0.130) and significant fibrosis (P = 0.066) between low normal and high normal ALT subgroups. While patients with PNALT were stratified by age of 40, significant necroinflammation (P = 0.578) and significant fibrosis (P = 1.000) were also comparable. (Table 2)

### Parameters associated with liver histological abnormalities

#### HBeAg-positive patients

In patients with PNALT, univariate analysis indicated that no parameter was associated with significant liver histological abnormality. In patients with elevated ALT, univariate analysis indicated age (P = 0.001), levels of platelet (PLT) (P < 0.001), PTA (P < 0.001), ALB (P = 0.003), ALT (P < 0.001), AST (P < 0.001) and HBV DNA (P < 0.001) were associated with significant necroinflammation, while age (P = 0.013), levels of PLT (P = 0.019), ALB (P = 0.003), ALT (P = 0.001), AST (P < 0.001) and HBV DNA (P < 0.001) were associated with significant fibrosis. The multivariate analysis of clinical parameters which independently associated with significant abnormality is shown in Table 3. Increasing age (P = 0.012), higher AST (P < 0.001) and lower HBV DNA (P < 0.001) were associated with significant necroinflammation, while higher AST (P < 0.001), lower HBV DNA (P = 0.004) and ALB (P = 0.027) were associated with significant fibrosis in patients with elevated ALT. 

**Table 3:** Association between histological significant abnormalites and different parameters by multivariate logistic regression.

		Parameter	OR	95% CI	P value
HBeAg-positive					
Elevated ALT (n = 431)	Grade≥A2	Age	1.042	1.009-1.076	0.012
		AST	1.015	1.008-1.022	<0.001
		HBV DNA	0.671	0.546-0.825	<0.001
	Stage≥F2	ALB	0.933	0.877-0.992	0.027
		AST	1.024	1.013-1.036	<0.001
		HBV DNA	0.685	0.530-0.885	0.004
HBeAg-negative					
Elevated ALT (n = 104)	Grade≥2	AST	1.021	1.005-1.038	0.009

ALT, alanine aminotransaferase; AST, aspirate aminotransferase; ALB, albumin; OR, odds ratio; CI, confidence interval

#### HBeAg-negative patients

In patients with PNALT, univariate analysis indicated that no parameter was associated with significant histological abnormality. In patients with elevated ALT, levels of PTA (P = 0.008), ALB (P = 0.026), ALT (P < 0.001), AST (P < 0.001) and HBV DNA (P = 0.013) were associated with significant necroinflammation, while levels of ALT (P = 0.010) and AST (P = 0.009) were associated with significant fibrosis. The multivariate analysis of clinical parameters which were independently associated with significant abnormalities is shown in Table 3. Only higher AST was associated with significant necroinflammation in patients with elevated ALT (P = 0.009).

## Discussion

Limited studies have been conducted on CHB patients with liver histology by a large population in China previously. Furthermore there are still many debates on what types of patients need liver biopsy, especially those with PNALT have not been evaluated clearly in previous researches. Six hundred and seventy-five patients were recruited in this study, all of whom were totally treatment-naive and in different phases of CHB [8]. The associations of various routinely available clinical parameters and liver histological abnormalities were also investigated.

Previous investigations indicated HBeAg-positive patients with PNALT usually have high level of HBV DNA with no or minimal liver histological changes [17–19]. As well as trials showed they tend to have no good response to current available antiviral therapy [20,21]. Thus liver biopsy or treatment is not recommended on these patients [8,9,22]. However the latest EASL guideline took a more active management that liver biopsy or even therapy should implement on immune tolerant patients over 30 years old [10]. Therefore we first examined the characteristics of histological changes in HBeAg-positive patients with PNALT. Although they did not have active inflammation biochemically, our results demonstrate significant fibrosis could be found in almost half of them (49.4%). Several reports from Asia on these same types of patients were consistent with ours. One study from Hong Kong found that 22.5% of patients had significant histological abnormalities [11]. Furthermore, a study from India showed 40.2% and another from Indonesia reported 68.6% of these patients had significant fibrosis [23,24]. So our results suggest a proportion of HBeAg-positive patients with PNALT have significant fibrosis, and the notion they are deemed as ‘healthy’ maybe incorrect in Chinese and Asian patients.

It has been proven that treatment in patients with advanced liver fibrosis can lower the risks of HCC and cirrhotic complications [25], so the next question is what kinds of HBeAg-positive patients with PNALT should have closer examination. We stratified them by age of 30 according to EASL’s opinion. Results present patients over 30 years old had a higher frequency of significant fibrosis than those under 30 (87.5% vs. 45.5%, P = 0.058), which narrowly failed to reach statistical significance possibly due to the small sample size in the older subgroup (n = 8). Considering no any biochemical parameters associated with significant fibrosis, therefore age over 30 years might be a risk factor in HBeAg-positive patients with PNALT. One study from Hong Kong approved us by indicating that the risk of liver fibrosis increases after age 35 in HBeAg-positive patients [26]. Nevertheless it concentrated on patients with advanced fibrosis (F3 - F4) by TE measurement instead of histological proof. Recently Zoulim illustrates one step beyond the major guidelines would be to start therapy in all patients with normal ALT who show relatively low levels of viremia, including patients in their 20s, not just those beyond 40s [27]. The author believes many unnecessary deaths may occur that might be prevented by earlier antiviral intervention, additionally considering its long duration, initiation and promotion may both be significant during the immune tolerant phase, increasing the risk of HCC later in life even in the absence of cirrhosis [27]. So liver biopsy or at least TE should be strongly indicated on immune tolerant patients who have favorable age, and a large prospective cohort study should be conducted to address the efficacy and cost-effectiveness of antiviral therapy for these patients with significant fibrosis in the prevention of liver cirrhosis and HCC.

We then investigated the characteristics of histological changes in HBeAg-negative patients with PNALT. There was a systemic review on these special patients to determine the prevalence of significant liver disease and its associating factors [28]. According to this review, histological significant liver disease was rare in these patients, who required neither liver biopsy nor immediate therapy but continued follow-up. There was less than 5% of significant fibrosis found in them in three European studies together [15,29,30]. However, studies from Japan and India indicated significant fibrosis accounted for 35% and 14% in these patients respectively [23,31]. There was 30.9% in our cohort, which is similar to the patients from other Asian countries. It is unethical and impossible to perform liver biopsy in every HBeAg-negative patient with PNALT even significant fibrosis is not rare, but there are no favorable age and associated clinical parameters. Previous study demonstrated early liver cirrhosis was not uncommon among HBeAg-negative patients with normal ALT, probably ranging from 7.1% (probable cirrhosis) to 22.8% (possible cirrhosis) by TE with high degree of certainty [32]. As well as Chen’s consideration HBeAg-negative patients with PNALT are still at risk for HCC and liver-related death [33], therefore TE has an advantage and should be considered to evaluate the severity of liver fibrosis periodically. But the beneficial of antiviral therapy for these patients with significant fibrosis should also be further studied in the prevention of end-stage liver disease. In summary, our data indicate significant fibrosis is not rare in Chinese CHB patients with PNALT, and much higher than those in European studies. This difference could be explained as patients in China acquire the infection perinatally, with liver injury starting early in life.

An Italian population study suggested lowering the ULN of ALT to 30 U/L for men and 19 U/L for women [34], while a Korean study suggested reducing the ULN of ALT to 33 U/L for men and 25 U/L for women [35]. Both studies challenged the traditional threshold ALT ULN of 40 U/L. Reasons supported changing the new ALT ULN as patients below a normal ALT of 40 U/L, but above the new criterion had more significant histological abnormalities than those under the new level criterion [11,36,37]. On contrary some studies did not agree [23,30,38]. Our research demonstrates no differences were found in histological abnormalities between low normal and high normal ALT subgroups in patients with PNALT. So there may be no need to decrease the ULN of ALT in Chinese patients.

There are different opinions on the relationship between level of ALT and liver histological abnormalities [11,12,14,23,24,39]. Our study shows after multivariate logistic regression analyze, instead of ALT, higher AST could predict histological significant abnormalities in HBeAg-positive patients with elevated ALT, and predict significant inflammation in HBeAg-negative patients with elevated ALT. Hence AST is much more specific than ALT in evaluating the histological severity of patients as reported elsewhere [11,23,39]. Furthermore, major guidelines suggested CHB patients with minimally raised ALT should consider liver biopsies or non-invasive fibrosis assessment. As significant fibrosis is very common in patients with ALT 1-2×ULN (69.8% in the HBeAg-positive group and 73.3% in the HBeAg-negative group) in our cohort, so patients with minimally raised ALT may start antiviral therapy directly if liver biopsy or TE is not available.

The relationship between the level of HBV DNA and liver histological change remains controversial. Though most observations indicated HBV DNA is positively correlated with severe histological changes [11,13,15,24], few researches showed they are not in correlation [14,37]. Our results demonstrate HBV DNA was negatively associated with significant necroinflammation and fibrosis in HBeAg-positive patients with elevated ALT. But HBV DNA was not associated with significant histological abnormalities in HBeAg-negative patients with elevated ALT, and Park et al reported a similar result [12]. One possible explanation for this maybe they have prolonged liver necroinflammation and progression of liver fibrosis, which persisted even after immune control took over at the time of assessment [32].

There are several limitations during the interpretation of our findings. First, this is a retrospective cross-sectional study mainly consisted of HBeAg-positive patients and could not exclude the possibility of referral bias. Because patients in immune clearance phase, especially those with HBeAg-negative, tend to have fluctuating ALT levels and receive liver functional protection therapy to alleviate biochemical activity in China. However, previous studies on CHB patients with liver histology from China did not take these considerations, which may result in recruiting patients with more active and severe fibrosis of liver disease. Hence, we excluded many screened subjects mostly consisted of HBeAg-negative patients due to this reason so as to reduce the possible bias (Figure 1). But our study is able to recruit a large population of almost 700 patients, who could be generalized to other CHB patients. Second, HBV genotype and quantification of HBsAg were not measured in most of our studied subjects for they may not be available in routine clinical practice until recent years. Finally, the number of patients with PNALT is relatively small and without follow-up after liver biopsy. However, there had rare researches on both HBeAg-positive and HBeAg-negative patients with PNALT strictly defined simultaneously before, and the number of HBeAg-positive patients with PNALT in our study (n = 85) is large. Additionally, there were no any risk factors of significant fibrosis or suspected liver cirrhosis in the included biopsied patients with PNALT. So the results are still convincible. Despite these limitations, our data provide some information on liver histological characteristics in Chinese CHB patients. 

In conclusion, significant fibrosis is not rare in Chinese patients with PNALT. Liver fibrosis assessment should be strongly considered in these patients, especially HBeAg-positive patients over 30 years old. CHB patients with minimally raised ALT may be recommended to start antiviral therapy if liver biopsy or non-invasive fibrosis assessment is not available.

## References

[1] Dienstag JL (2008) Hepatitis B virus infection. N Engl J Med 359: 1486-1500. doi:10.1056/NEJMra0801644. PubMed: 18832247. 18832247

[2] Yuen MF, Hou JL, Chutaputti A (2009) Hepatocellular carcinoma in the Asia pacific region. J Gastroenterol Hepatol 24: 346-353. doi:10.1111/j.1440-1746.2009.05784.x. PubMed: 19220670. 19220670

[3] Lavanchy D (2004) Hepatitis B virus epidemiology, disease burden, treatment, and current and emerging prevention and control measures. J Viral Hepat 11: 97-107. doi:10.1046/j.1365-2893.2003.00487.x. PubMed: 14996343. 14996343

[4] Mani H, Kleiner DE (2009) Liver biopsy findings in chronic hepatitis B. Hepatology 49: S61-S71. doi:10.1002/hep.22930. PubMed: 19399798. 19399798

[5] Fung J, Lai CL, Seto WK, Yuen MF (2011) The use of transient elastography in the management of chronic hepatitis B. Hepatol Int 5: 868-875. doi:10.1007/s12072-011-9288-5. PubMed: 21695588. 21695588 PMC3215876

[6] ter Borg F, ten KF, Cuypers HT, Leentvaar-Kuijpers A, Oosting J et al. (2000) A survey of liver pathology in needle biopsies from HBsAg and anti-HBe positive individuals. J Clin Pathol 53: 541-548. doi:10.1136/jcp.53.7.541. PubMed: 10961179. 10961179 PMC1731225

[7] Regev A, Berho M, Jeffers LJ, Milikowski C, Molina EG et al. (2002) Sampling error and intraobserver variation in liver biopsy in patients with chronic HCV infection. Am J Gastroenterol 97: 2614-2618. doi:10.1111/j.1572-0241.2002.06038.x. PubMed: 12385448. 12385448

[8] Liaw Y, Kao J, Piratvisuth T, Chan H, Chien R et al. (2012) Asian-Pacific consensus statement on the management of chronic hepatitis B: a 2012 update. Hepatol Int 6: 531-561. doi:10.1007/s12072-012-9365-4. 26201469

[9] Lok AS, McMahon BJ (2009) Chronic hepatitis B: update 2009. Hepatology 50: 661-662. doi:10.1002/hep.23190. PubMed: 19714720. 19714720

[10] European Association for The Study of The Liver (2012) EASL clinical practice guidelines: Management of chronic hepatitis B virus infection. J Hepatol 57: 167-185. PubMed: 22436845. 22436845 10.1016/j.jhep.2012.02.010

[11] Seto WK, Lai CL, Ip PP, Fung J, Wong DK et al. (2012) A large population histology study showing the lack of association between ALT elevation and significant fibrosis in chronic hepatitis B. PLOS ONE 7: e32622. doi:10.1371/journal.pone.0032622. PubMed: 22389715. 22389715 PMC3289659

[12] Park JY, Park YN, Kim DY, Paik YH, Lee KS et al. (2008) High prevalence of significant histology in asymptomatic chronic hepatitis B patients with genotype C and high serum HBV DNA levels. J Viral Hepat 15: 615-621. doi:10.1111/j.1365-2893.2008.00989.x. PubMed: 18573162. 18573162

[13] Chan HL, Tsang SW, Liew CT, Tse CH, Wong ML et al. (2002) Viral genotype and hepatitis B virus DNA levels are correlated with histological liver damage in HBeAg-negative chronic hepatitis B virus infection. Am J Gastroenterol 97: 406-412. doi:10.1111/j.1572-0241.2002.05478.x. PubMed: 11866280. 11866280

[14] Shao J, Wei L, Wang H, Sun Y, Zhang LF et al. (2007) Relationship between hepatitis B virus DNA levels and liver histology in patients with chronic hepatitis B. World J Gastroenterol 13: 2104-2107. PubMed: 17465456. 17465456 10.3748/wjg.v13.i14.2104 PMC4319133

[15] Zacharakis G, Koskinas J, Kotsiou S, Tzara F, Vafeiadis N et al. (2008) The role of serial measurement of serum HBV DNA levels in patients with chronic HBeAg(-) hepatitis B infection: association with liver disease progression. A prospective cohort study. J Hepatol 49: 884-891. doi:10.1016/j.jhep.2008.06.009. PubMed: 18674840. 18674840

[16] Bedossa P, Poynard T (1996) An algorithm for the grading of activity in chronic hepatitis C. The METAVIR Cooperative Study Group. Hepatology 24: 289-293. doi:10.1002/hep.510240201. PubMed: 8690394. 8690394

[17] Andreani T, Serfaty L, Mohand D, Dernaika S, Wendum D et al. (2007) Chronic hepatitis B virus carriers in the immunotolerant phase of infection: histologic findings and outcome. Clin Gastroenterol Hepatol 5: 636-641. doi:10.1016/j.cgh.2007.01.005. PubMed: 17428739. 17428739

[18] Hui CK, Leung N, Yuen ST, Zhang HY, Leung KW et al. (2007) Natural history and disease progression in Chinese chronic hepatitis B patients in immune-tolerant phase. Hepatology 46: 395-401. doi:10.1002/hep.21724. PubMed: 17628874. 17628874

[19] Liaw YF, Chu CM (2009) Hepatitis B virus infection. Lancet 373: 582-592. doi:10.1016/S0140-6736(09)60207-5. PubMed: 19217993. 19217993

[20] Perrillo RP, Lai CL, Liaw YF, Dienstag JL, Schiff ER et al. (2002) Predictors of HBeAg loss after lamivudine treatment for chronic hepatitis B. Hepatology 36: 186-194. doi:10.1016/S0168-8278(02)80657-2. PubMed: 12085364. 12085364

[21] Lau GK, Piratvisuth T, Luo KX, Marcellin P, Thongsawat S et al. (2005) Peginterferon Alfa-2a, lamivudine, and the combination for HBeAg-positive chronic hepatitis B. N Engl J Med 352: 2682-2695. doi:10.1056/NEJMoa043470. PubMed: 15987917. 15987917

[22] Han K, Kim D (2008) Chronic HBV infection with persistently normal ALT b. not to treat. Hepatol Int 2: 185-189. doi:10.1007/s12072-008-9068-z.

[23] Kumar M, Sarin SK, Hissar S, Pande C, Sakhuja P et al. (2008) Virologic and histologic features of chronic hepatitis B virus-infected asymptomatic patients with persistently normal ALT. Gastroenterology 134: 1376-1384. doi:10.1053/j.gastro.2008.02.075. PubMed: 18471514. 18471514

[24] Lesmana CR, Gani RA, Hasan I, Simadibrata M, Sulaiman AS et al. (2011) Significant hepatic histopathology in chronic hepatitis B patients with serum ALT less than twice ULN and high HBV-DNA levels in Indonesia. J Dig Dis 12: 476-480. doi:10.1111/j.1751-2980.2011.00540.x. PubMed: 22118698. 22118698

[25] Liaw YF, Sung JJ, Chow WC, Farrell G, Lee CZ et al. (2004) Lamivudine for patients with chronic hepatitis B and advanced liver disease. N Engl J Med 351: 1521-1531. doi:10.1056/NEJMoa033364. PubMed: 15470215. 15470215

[26] Wong GL, Wong VW, Choi PC, Chan AW, Chim AM et al. (2009) Clinical factors associated with liver stiffness in hepatitis B e antigen-positive chronic hepatitis B patients. Clin Gastroenterol Hepatol 7: 227-233. doi:10.1016/j.cgh.2008.10.023. PubMed: 19121647. 19121647

[27] Zoulim F, Mason WS (2012) Reasons to consider earlier treatment of chronic HBV infections. Gut 61: 333-336. doi:10.1136/gutjnl-2012-302514d.89. PubMed: 22147510. 22147510

[28] Papatheodoridis GV, Manolakopoulos S, Liaw YF, Lok A (2012) Follow-up and indications for liver biopsy in HBeAg-negative chronic hepatitis B virus infection with persistently normal ALT: a systematic review. J Hepatol 57: 196-202. doi:10.1016/j.jhep.2011.11.030. PubMed: 22450396. 22450396

[29] Martinot-Peignoux M, Boyer N, Colombat M, Akremi R, Pham BN et al. (2002) Serum hepatitis B virus DNA levels and liver histology in inactive HBsAg carriers. J Hepatol 36: 543-546. doi:10.1016/S0168-8278(02)00004-1. PubMed: 11943427. 11943427

[30] Papatheodoridis GV, Manesis EK, Manolakopoulos S, Elefsiniotis IS, Goulis J et al. (2008) Is there a meaningful serum hepatitis B virus DNA cutoff level for therapeutic decisions in hepatitis B e antigen-negative chronic hepatitis B virus infection? Hepatology 48: 1451-1459. doi:10.1002/hep.22518. PubMed: 18924246. 18924246

[31] Ikeda K, Arase Y, Saitoh S, Kobayashi M, Someya T et al. (2006) Long-term outcome of HBV carriers with negative HBe antigen and normal aminotransferase. Am J Med 119: 977-985. doi:10.1016/j.amjmed.2006.04.036. PubMed: 17071167. 17071167

[32] Wong GL, Wong VW, Choi PC, Chan AW, Chim AM et al. (2008) Evaluation of alanine transaminase and hepatitis B virus DNA to predict liver cirrhosis in hepatitis B e antigen-negative chronic hepatitis B using transient elastography. Am J Gastroenterol 103: 3071-3081. doi:10.1111/j.1572-0241.2008.02157.x. PubMed: 19086958. 19086958

[33] Chen JD, Yang HI, Iloeje UH, You SL, Lu SN et al. (2010) Carriers of inactive hepatitis B virus are still at risk for hepatocellular carcinoma and liver-related death. Gastroenterology 138: 1747-1754. doi:10.1053/j.gastro.2010.01.042. PubMed: 20114048. 20114048

[34] Prati D, Taioli E, Zanella A, Della TE, Butelli S et al. (2002) Updated definitions of healthy ranges for serum alanine aminotransferase levels. Ann Intern Med 137: 1-10. doi:10.7326/0003-4819-137-8-200210150-00027-w1. PubMed: 12093239. 12093239

[35] Lee JK, Shim JH, Lee HC, Lee SH, Kim KM et al. (2010) Estimation of the healthy upper limits for serum alanine aminotransferase in Asian populations with normal liver histology. Hepatology 51: 1577-1583. doi:10.1002/hep.23505. PubMed: 20162730. 20162730

[36] Assy N, Beniashvili Z, Djibre A, Nasser G, Grosovski M et al. (2009) Lower baseline ALT cut-off values and HBV DNA levels better differentiate HBeAg- chronic hepatitis B patients from inactive chronic carriers. World J Gastroenterol 15: 3025-3031. doi:10.3748/wjg.15.3025. PubMed: 19554656. 19554656 PMC2702111

[37] Lai M, Hyatt BJ, Nasser I, Curry M, Afdhal NH (2007) The clinical significance of persistently normal ALT in chronic hepatitis B infection. J Hepatol 47: 760-767. doi:10.1016/j.jhep.2007.07.022. PubMed: 17928090. 17928090

[38] Tai DI, Lin SM, Sheen IS, Chu CM, Lin DY et al. (2009) Long-term outcome of hepatitis B e antigen-negative hepatitis B surface antigen carriers in relation to changes of alanine aminotransferase levels over time. Hepatology 49: 1859-1867. doi:10.1002/hep.22878. PubMed: 19378345. 19378345

[39] Hui AY, Chan HL, Wong VW, Liew CT, Chim AM et al. (2005) Identification of chronic hepatitis B patients without significant liver fibrosis by a simple noninvasive predictive model. Am J Gastroenterol 100: 616-623. doi:10.1111/j.1572-0241.2005.41289.x. PubMed: 15743360. 15743360

